# Dynamics of *Microcystis* surface scum formation under different wind conditions: the role of hydrodynamic processes at the air-water interface

**DOI:** 10.3389/fpls.2024.1370874

**Published:** 2024-03-11

**Authors:** Huaming Wu, Xingqiang Wu, Lorenzo Rovelli, Andreas Lorke

**Affiliations:** ^1^ Institute for Environmental Sciences, University of Kaiserslautern-Landau (RPTU), Landau, Germany; ^2^ Key Laboratory of Algal Biology of Chinese Academy of Sciences, Institute of Hydrobiology, Chinese Academy of Sciences, Wuhan, China; ^3^ Now at the Department of Ecology, Federal Institute of Hydrology - BfG, Koblenz, Germany

**Keywords:** aquatic ecosystems, cyanobacterial blooms, wind disturbance, surface tension, biological-physical interactions, capillary force

## Abstract

Due to climate change, *Microcystis* blooms occur at increasing frequencies in aquatic ecosystems worldwide. Wind-generated turbulence is a crucial environmental stressor that can vertically disperse the *Microcystis* surface scum, reducing its light availability. Yet, the interactions of *Microcystis* scum with the wind-generated hydrodynamic processes, particularly those at the air-water interface, remain poorly understood. Here, we explore the response of *Microcystis* (including colony size and migration dynamics) to varying magnitudes and durations of intermittent wind disturbances in a mesocosm system. The flow velocities, size of *Microcystis* colonies, and the areal coverage of the water surface by scum were measured through video observations. Our results demonstrate that low wind speeds increase colony size by providing a stable condition where *Microcystis* forms a scum layer and aggregates into large colonies at the air-water interface. In contrast, wind disturbances disperse scum and generate turbulence, resulting in smaller colonies with higher magnitudes of wind disturbance. We observed that surface scum can form rapidly following a long period (6 h) of high-magnitude (4.5 m s^-1^) wind disturbance. Furthermore, our results indicate reduced water surface tension caused by the presence of *Microcystis*, which can decrease surface flow velocity and counteract wind-driven mixing. The reduced surface tension may also drive lateral convection at the water surface. These findings suggest that *Microcystis* reduces surface tension, likely by releasing surface-active materials, as an adaptive response to various wind conditions. This could result in an increased rate of surface scum re-formation under wind conditions and potentially facilitate the lateral expansion of scum patches during weak wind periods. This study reveals new insights into how *Microcystis* copes with different wind conditions and highlights the importance of the air-water interface for *Microcystis* scum dynamics.

## Highlights

- Larger colony size of *Microcystis* at low wind speed
- Rapid formation of scum after strong wind disturbances
- Increasing re-formation rate of surface scum during recurring disturbances
- Increasing biomass of *Microcystis* leads to reduction of water surface tension
- Reduced surface tension can be advantageous for *Microcystis* surface scum

## 1 Introduction

Cyanobacterial blooms have been becoming a globally relevant threat to the ecological integrity of inland and coastal waters (Huisman et al., 2018; Ho et al., 2019). As one of the most common and ubiquitous cyanobacterial genera, *Microcystis* spp. can float upward to the water surface and form dense surface scum, i.e., visible mucilaginous cyanobacteria accumulating at the water surface (Oliver, 1994), disrupting the functioning of aquatic ecosystems.

Buoyancy regulation is an important cellular feature that enables *Microcystis* spp. to maintain their position at the water surface and form surface scum (Reynolds, 1987; Oliver, 1994). The buoyancy of *Microcystis* can be modulated by (i) the carbon-reserve metabolism, i.e., the accumulation of intracellular carbohydrates (Visser et al., 1997), (ii) the synthesis and collapse of gas-vesicles in the cells (Dunton and Walsby, 2005), (iii) the formation of colonies, i.e., aggregations of *Microcystis* cells embedded in a mucilaginous matrix, and (iv) trapping of gas bubbles within the colonies (Dervaux et al., 2015; Aparicio Medrano et al., 2016). The colony size of *Microcystis* is considered an important factor for surface scum formation as their floatation velocity increases with increasing colony size (Xiao et al., 2018; Wu et al., 2020).

Disturbances induced by wind are among the most important stressors that counteract scum formation (George and Edwards, 1976). Wind-generated turbulence can vertically disperse surface scum to deeper depths and reduce the light availability for *Microcystis* when the wind speed exceeds a critical value (2.6-3 m s^-1^) (Cao et al., 2006; Wu et al., 2015). Below the critical wind speed, wind-generated flow leads to the accumulation of *Microcystis* in downwind areas of the basin (Chen et al., 2023). Although the flows generated by wind, including basin-scale circulation, waves and turbulent eddies exceed the size of *Microcystis* colonies by far, these motions are coupled through the turbulent cascade to the small-scale viscous environment of cells and colonies (Regel et al., 2004). Although wind disturbances at appropriate magnitudes can promote the aggregation of *Microcystis* colonies, continuous wind disturbances act as stressors by inhibiting growth and their aggregation through shear forces (O’Brien et al., 2004; Liu et al., 2019; Zhao et al., 2020).

The above studies have primarily focused on bulk water, without considering the presence of a free-water surface, i.e., the air-water interface. The physicochemical and biological properties at the water surface are measurably distinct from those in the underlying water, and various physical processes, such as momentum transfer from wind to water, wind-generated wave, and capillary effect can occur (Vella and Mahadevan, 2005; Cunliffe et al., 2013). For example, current knowledge focuses on how wind-generated turbulence in the water column affects colony size dynamics, but often overlooks the frequent aggregation of *Microcystis* at the water surface during low wind periods. This is due to a lack of understanding of aggregation mechanisms at the water surface and their role in colony size dynamics. Neglecting the aggregation of colonies at the water surface may lead to an overestimation of the role of colony size in surface scum formation, as larger colonies in the epilimnion are often considered as a cause (Zhu et al., 2014), instead of being the consequence of surface scum formation.

Current understandings of the interactions between *Microcystis* and wind-generated hydrodynamic processes are largely one-way, neglecting the potential feedback of *Microcystis* on hydrodynamics. Studies have revealed that phytoplankton can affect the physical properties of water; Dervaux et al. (2015) observed non-Newtonian behavior of algal suspensions at low shear stress, with viscosity increasing by three orders of magnitude. Additionally, proteins extracted from algae can reduce interfacial tension at the air-water interface, even at relatively low bulk concentrations (Chronakis et al., 2000). They attributed these findings to the release of extracellular polymeric substances (EPSs) by phytoplankton. The alteration in physical properties of water resulting from the released EPSs could be a response of phytoplankton to stressors, constituting two-way interactions between *Microcystis* and wind-generated hydrodynamic processes. However, these interactions have rarely been studied.

As a result of climate change, wind speeds are expected to decrease in some regions under future climates (Vautard et al., 2010; Ranjbar et al., 2022), necessitating the testing of how *Microcystis* responds to different magnitudes and durations of intermittent wind disturbances. In this study, we conducted laboratory experiments in annular flumes, in which wind-driven flow was simulated by controlled air circulation above the water surface. Different magnitudes of wind forcing (0.5, 1.5, 3.8, and 4.5 m s^-1^) and durations of their periodic occurrence (3 and 6 h) were used to simulate the periodic formation, development, dissipation and re-formation of surface scum over a period of seven days. We hypothesized that *Microcystis* surface scum can interact with the hydrodynamic processes at the air-water interface by affecting the physical properties of water, i.e., water surface tension. The experiments aimed at (i) studying the response of colony size and surface scum dynamics of *Microcystis* to wind-generated turbulence, and (ii) exploring the effect of *Microcystis* surface scum on hydrodynamic processes mediated by surface tension. The results of this study are expected to be instrumental in the mechanistic and process-based understanding of surface scum dynamics.

## 2 Materials and methods

### 2.1 Source of material

A stock of phytoplankton (approximately 90% of the phytoplankton was *Microcystis aeruginosa* by microscopic observation) was collected from the Moselle River in southwest Germany on 9 August 2022 during a heavy *Microcystis* bloom. Colonies were collected from the water surface using a silk plankton net with a 40 μm mesh size. To select predominantly *Microcystis* colonies, the samples were first filtered through a 500 μm sieve to remove large particles and then through a 40 μm sieve. The filtered *Microcystis* colonies with sizes between 40 and 500 μm were stored and cultured at 20 ± 1°C and photosynthetically active radiation (PAR) of 15 μmol photons s^−1^ m^−2^.

### 2.2 Experimental design

#### 2.2.1 Flume experiments

The dynamics of *Microcystis* colonies under different wind conditions were studied in five annular flumes with outer and inner diameters of 700 and 560 mm (**Supplementary Figure S1**). The light intensity and wind speed were varied in individual flumes. Flow velocities and *Microcystis* colonies were observed in video recordings with digital cameras (Raspberry Pi HQ Camera, United Kingdom, 1080p, 30 fps) at the water surface and at three different depths (near the water surface at 0 – 6 cm depth, in a middle layer at 12 – 18 cm depth and close to the flume bottom at 24 – 30 cm depth). All operations, including environmental settings (i.e., wind and light) and video recordings (i.e., cameras and laser light sheet for underwater illumination) were fully automated and computer-controlled (see **Supplementary Figure S1** for details).

Before the experiment, all flumes were sterilized for 30 min using an ozone generator. The flumes were then filled with distilled water (volume: ~ 41.5 L, water height: ~ 30 cm) before a nutrient stock solution (BG-11, see **Supplementary Table S1** for details) and *Microcystis* colonies were added to the five flumes. The final concentration of nutrients in the flumes was 10% (v/v) of BG-11 medium. Optical density at a wavelength of 680 nm (OD_680_) was used as a proxy for *Microcystis* biomass (Lv et al., 2018; Wu et al., 2019) and measured by a spectrophotometer (Novaspec II, Amersham Pharmacia Biotech Inc, UK). The initial optical density (at 680 nm) of *Microcystis* in the flumes was 0.05 ± 0.01, which corresponds to an approximate cell density of 7.9 ×10^5^ cells mL^-1^ (Lv et al., 2018).

**Figure 1:**
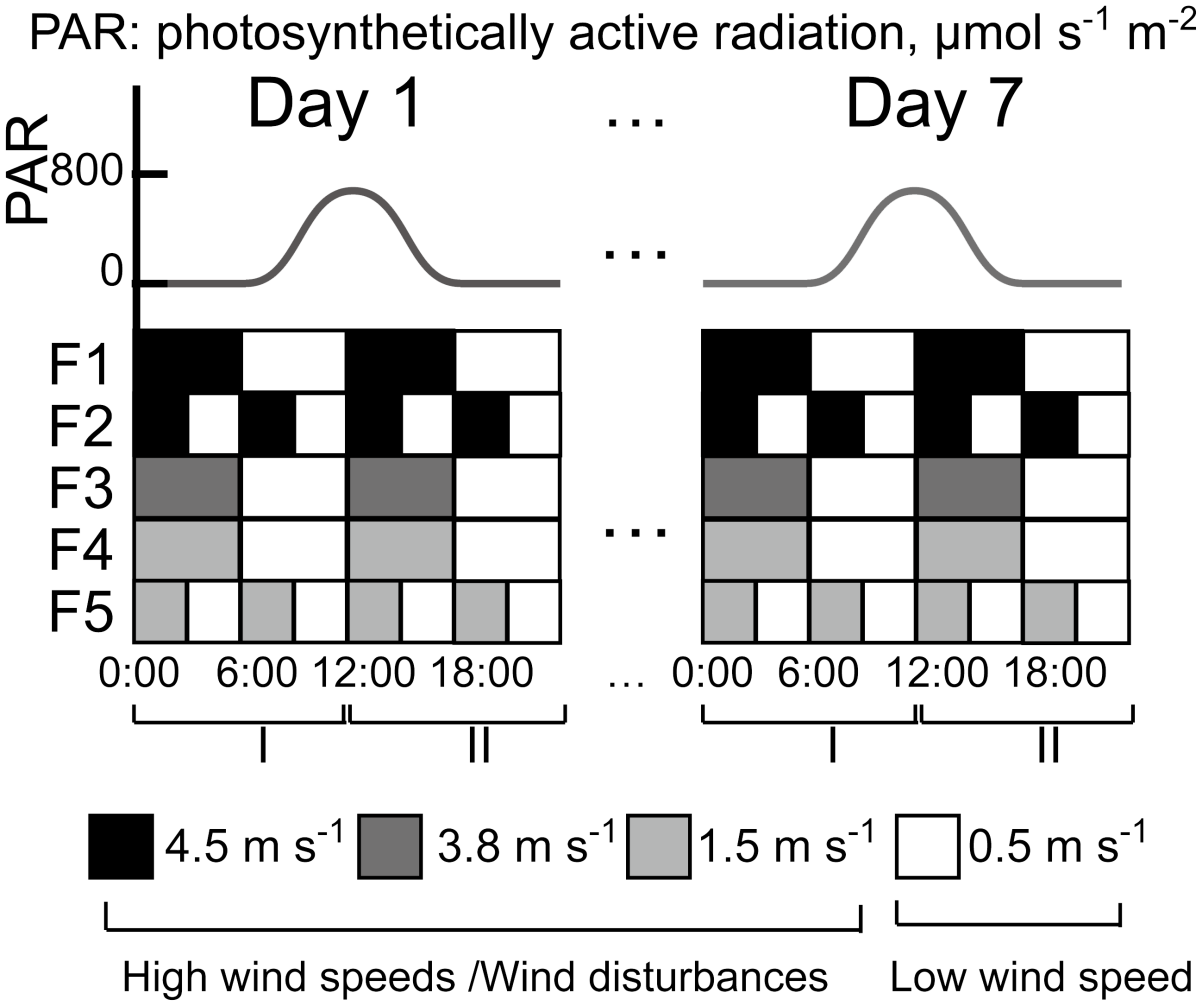
Diurnal variations of photosynthetically active radiation (PAR, upper panel) and wind speed (lower panel) in the five flumes (F1 – F5) during the 7-day experiment. The morning period (0:00 to 12:00) was defined as stage I and the afternoon (12:00 to 24:00) was defined as stage II. The wind speeds of 4.5, 3.8 and 1.5 m s^-1^ were defined as high wind speeds and the wind speed of 0.5 m s^-1^ was defined as low wind speed.

During the following 7-day experimental period, different magnitudes (0.5, 1.5, 3.8, 4.5 m s^-1^) and durations (3 and 6 h) of intermittent wind forcing were applied in the flumes at a constant water temperature of 22.3 ± 1.0°C. The chosen temperature is within the range of water temperatures for which *Microcystis* blooms were observed under field conditions (18 - 24°C, Feng et al., 2019). No temperature difference between the water surface, the middle layer and the bottom of the flume was observed. Two different wind forcing (high wind speed and low wind speed) were applied alternately for two different durations. The wind speed was measured 2 cm above the water surface at a location between the two fans using a hot-wire anemometer (Testo 425, Germany). The high wind speed in the flumes F1 – F5 was 4.5, 4.5, 3.8, 1.5, and 1.5 m s^-1^, respectively, whereas a wind speed of 0.5 m s^-1^ was used in all flumes during the low wind periods (**Figure 1**). The high wind speed periods were defined as wind disturbances. Due to **a** technical issue, the experiment in F4 started at 11:00 on Day 2. Surface waves were observed in our experiment during wind disturbances; the amplitudes of the waves were determined to be < 8 mm through visual estimates using video footage. Due to the differing aerodynamic characteristics between the flume and the atmosphere, it is not easy to directly compare the airflow generated in the flumes with wind velocities measured in the atmospheric boundary layer above water surfaces. We estimated an equivalent wind speed in the atmospheric boundary layer at a standard height of 10 m (*U*_10_) from observed surface flow velocities (*U*_0_, described below) using a fixed wind factor (the ratio of wind speed at 10 m to the surface flow velocity of water, *f* = 0.01 [Wu, 1975)] as *U*_10_=*U*_0_/*f*.

We applied a diurnal light cycle (12:12 h light: dark periods) to all flumes. To simulate the light conditions in the field, the irradiance at the water surface was modulated as a sinusoidal function with a maximum irradiance of 800 μmol photons m^-2^ s^−1^ and truncated to zero for the nights (18:00 – 6:00) in induvial flumes (**Figure 1**). We defined the period 0:00 – 12:00 as stage I and the period 12:00 – 24:00 as stage II.

Videos were recorded at hourly intervals for estimating the surface flow velocity and areal coverage of the water surface with *Microcystis*. In addition, flow velocities and size of *Microcystis* colonies were observed at three different depths in the bulk water. The biomass of *Microcystis* colonies at the water surface and in the bulk water (~15 cm depth) were daily measured during 9:00- 11:00 am (low wind periods).

#### 2.2.2 Supplementary experiment

Due to the sampling difficulties caused by strong heterogeneity in the surface scum layer and dispersal of surfactant molecules during sampling, surface tension measurements could not be conducted under the prevailing flow conditions in the flume experiment. Consequently, we conducted an additional experiment to investigate the relationship between surface tension and *Microcystis* biomass. We measured the OD_680_ (as described above) and surface tension (using the Wilhelmy plate method with a tensiometer (TC1, Lauda Scientific, Germany)) of various *Microcystis* samples at different dilution factors (0%,1.5%, 3%, 6%, 9%, 15%, 75% and 100%). To ensure the OD_680_ did not exceed the detection limit of the spectrophotometer, the OD_680_ of algal samples higher than 0.3 were diluted 10 – 50 times, and then the OD_680_ of algal samples was calculated from OD_680_ of the diluted samples multiplied by the dilution factor.

### 2.3 Parameters of *Microcystis* and hydrodynamics measurements

Time-resolved observations of *Microcystis* colonies were obtained from video observations of the water surface using a down-looking digital camera (for the observation of surface scum and velocities of *Microcystis*) and of the bulk water (at depths of 0 – 4 cm, 12 – 16 cm and 26 – 30 cm) using side-looking cameras (for the observation of colony size and velocities of *Microcystis*). Videos of 90 s duration were hourly recorded. During the recordings at the water surface, illumination was provided by an LED spotlight, while during the recordings of the side-looking cameras, illumination in the bulk water was provided by a vertically oriented laser light sheet, which illuminated a central plane of the flow channel using a continuous-wave line laser (InLine HP, MediaLas, Germany; output power: 500 mW, green, 532 nm). Colonies within the approximately 3 mm thick light sheet were observed using three digital cameras in a perpendicular arrangement (**Supplementary Figure S1**). The resolution and field of view of the videos recorded by the cameras used in the experiments are shown in **Supplementary Table S2**.

Individual *Microcystis* colonies were detected in each image based on intensity thresholds during automated image processing. The analysis provided surface areal coverage of *Microcystis* (SAC, calculated as the percentage of water surface that was covered by colonies) for down-looking cameras, the equivalent spherical diameter of colonies for side-looking cameras, as well as colony velocities estimated using particle tracking velocimetry (Kelley and Ouellette, 2011). We estimated and summed up the area covered by the colonies for each frame, which was then divided by the camera’s field of view to calculate the SAC. The change rate of surface areal coverage (dSAC/dt) during low wind periods, representing the reformation rate of surface scum, was estimated by the difference of mean SAC between subsequent recordings. The volumetric median colony diameter (*D_v_*_50_, i.e., the colony size corresponding to the 50^th^ percentile of observed colony volumes) was used to characterize the average colony size as described in Wu et al. (2019) and Wu et al. (2020). More than 300 colonies on each of the videos were used to estimate *D_v_*_50_. The rate of change of colony size (d*D_v_*_50_/dt) was used to characterize the aggregation or disaggregation of colonies. Since surface scum formed during low wind periods and was dispersed during wind disturbances, the *D_v_*_50_ near the water surface at the beginning of a wind disturbance, when the surface scum layer was dispersed, was considered as a representative colony size at the water surface. To compare the colony size dynamics at the water surface and in the bulk water, d*D_v_*_50_/dt in the bulk water was estimated as the difference of mean *D_v_*_50_ near the water surface during wind disturbances between subsequent recordings. The d*D_v_*_50_/dt at the water surface was estimated by the ratio of the difference of mean *D_v_*_50_ near the water surface between the beginning of the wind disturbance and the end of the preceding wind disturbance to the duration of the wind disturbance.

The mean velocities of colonies at the water surface and in the bulk water were estimated by observing the displacement of identified colonies via particle tracking velocimetry, using the predictive tracking algorithm described by Kelley and Ouellette (2011).

We did not observe a consistent size-dependence of colony velocities (**Supplementary Figure S2**) and therefore considered the observed colony velocities as a proxy for flow velocity.

We use root-mean-square velocity fluctuations (*U_rms_*) as the measure of the intensity of turbulence, which scales with the square root of turbulence kinetic energy, and is calculated as:


(1) $$U_{rms}=\sqrt{\frac{1}{2}\left(\bar{{\left(u^{\prime }\right)}^{2}}+\bar{{\left(v^{\prime }\right)}^{2}}\right)}$$

where the fluctuating velocity components of the horizontal (*u’*) and vertical (*v’*) flow velocities are the difference between the instantaneous and the temporarily averaged velocities (Reynolds decomposition: $u'=u-\bar{u}$ and $v'=v-\bar{v}$ ). The temporarily averaged velocity was calculated by averaging the velocities of all colonies in each video.

### 2.4 Statistical analyses

Shapiro-Wilk test was used to assess the normality of data. A *post hoc* LSD (least significant difference) test with one-way ANOVA was used to compare the surface areal coverage (SAC) and the flow velocity during low wind periods at different depths at both stages among the different flumes if the data were normally distributed with homogeneous variance, otherwise, Kruskal-Wallis tests were used. The differences in *D_v50_* of colonies among different flumes as well as the difference between the rate of colony size change (d*D_v50_*/dt) at the water surface and in the bulk water were compared using the student *t*-test. The relationships between mean SAC and *D_v50_*, mean SAC and d*D_v50_*/d*t*, normalized flow velocity and SAC as well as between rate of change in SAC and time were fitted by linear regressions. All statistical analyses were performed using the software package SPSS 27.0 (IBM Corp, USA). Data are presented as mean ± standard deviation and were tested for statistical significance at a significance level (*p*) of 0.05 unless stated otherwise.

## 3 Results

### 3.1 Dynamics of *Microcystis* surface scum formation

**Figure 2:**
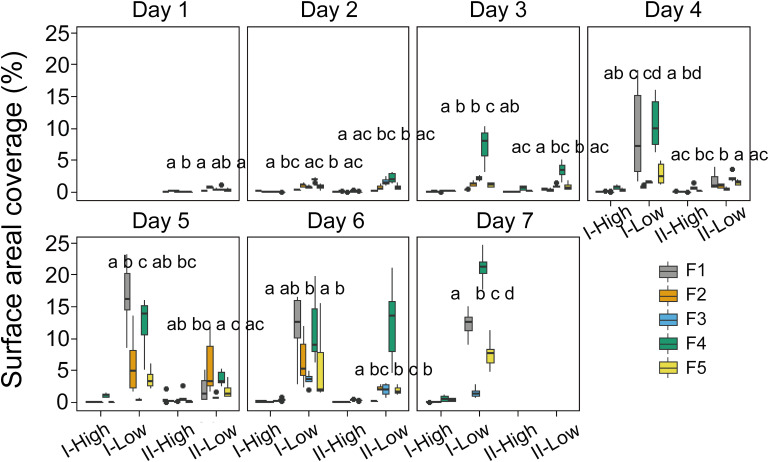
Temporal dynamics of the surface areal coverage of *Microcystis* colonies in different stages (I and II) and experimental flumes (F1 – F5, see legend for color assignment). High and Low represent the periods of high wind speed (wind disturbances, 4.5 m s^-1^ for F1 and F2, 3.8 m s^-1^ for F3, 1.5 m s^-1^ for F4 and F5) and low wind speed (0.5 m s^-1^), respectively. Different panels denote the time (day) after the start of the experiment. Each box plot shows the mean surface areal coverage observed in the hourly video observations (*n*=6). Different letters on top of the box plots indicate significant differences (*p* < 0.05) in surface areal coverage during low wind periods between different groups at each stage, while the same or no letter indicates no significant difference (Kruskal-Wallis tests). F1: 6 h of high-magnitude (4.5 m^-1^) wind disturbance; F2: 3 h of high-magnitude wind disturbance; F3: 6 h of moderate-magnitude (3.8 m^-1^) wind disturbance; F4: 6 h of low-magnitude (1.5 m^-1^) wind disturbance; F5: 3 h of low-magnitude wind disturbance.

**Figure 3:**
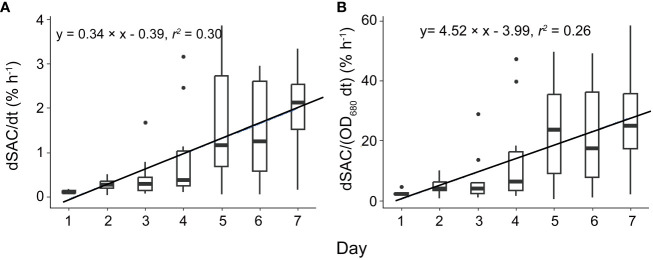
The temporal change of the rate of change in scum areal coverage (**A**, dSAC/dt) and the rate of change in scum areal coverage per unit biomass [**B**, dSAC/(OD_680_ dt)]. Significant linear relationships between dSAC/dt as well as dSAC/(OD_680_ dt) and time were observed (*p* < 0.05). Each box plot shows the dSAC/dt or dSAC/(OD_680_ dt). Both solid lines represent linear regressions based on the equations provided in the legend, with a significance level of *p* < 0.05. F1: 6 h of high-magnitude (4.5 m^-1^) wind disturbance; F2: 3 h of high-magnitude wind disturbance; F3: 6 h of moderate-magnitude (3.8 m^-1^) wind disturbance; F4: 6 h of low-magnitude (1.5 m^-1^) wind disturbance; F5: 3 h of low-magnitude wind disturbance.

Surface scum layers developed during low wind periods and disappeared during wind disturbance over the seven-day experimental period (**Figure 2**). During wind disturbances (1.5 – 4.5 m s^-1^), the areal coverage of the water surface with *Microcystis* colonies was generally < 1%, whereas it increased during low wind period (0.5 m s^-1^) gradually from 0.1 to 25% from Day 1 to Day 7. The observed rates of change in SAC (dSAC/dt) and rates of change in SAC per unit biomass (dSAC/(OD_680_ dt)) during low wind speed linearly increased over time (*p* < 0.05, **Figure 3**), suggesting that the surface scum recovered from wind disturbances at increasing rates. Similar to surface coverage, the biomass of *Microcystis* colonies in the thin surface layer showed a 2 - 6 fold increase from Day 1 to Day 7 (**Supplementary Figure S3A**).

The formation of surface scum followed a diurnal pattern with the average surface coverage during low wind periods in the afternoon (stage II) being reduced by 87.7%, 36.6%, 31.3%, 44.5% and 51.1% (for F1 – F5) in comparison to that in the morning (stage I) (**Figure 2**).

The dynamics of surface scum formation varied in dependence on wind conditions (**Figure 2**). The prolonged duration of low wind periods promoted scum formation: the surface coverage in flumes F1 and F4 was significantly higher than that in F2 and F5 in most cases (*p* < 0.05). Wind magnitudes of 4.5 and 1.5 m s^-1^ promoted the subsequent surface scum formation, in comparison to the intermediate magnitude of 3.8 m s^-1^: In most cases, the surface areal coverage in F1 and F4 was higher than that in F3 (*p* < 0.05).

### 3.2 Dynamics of *Microcystis* colony size

**Figure 4:**
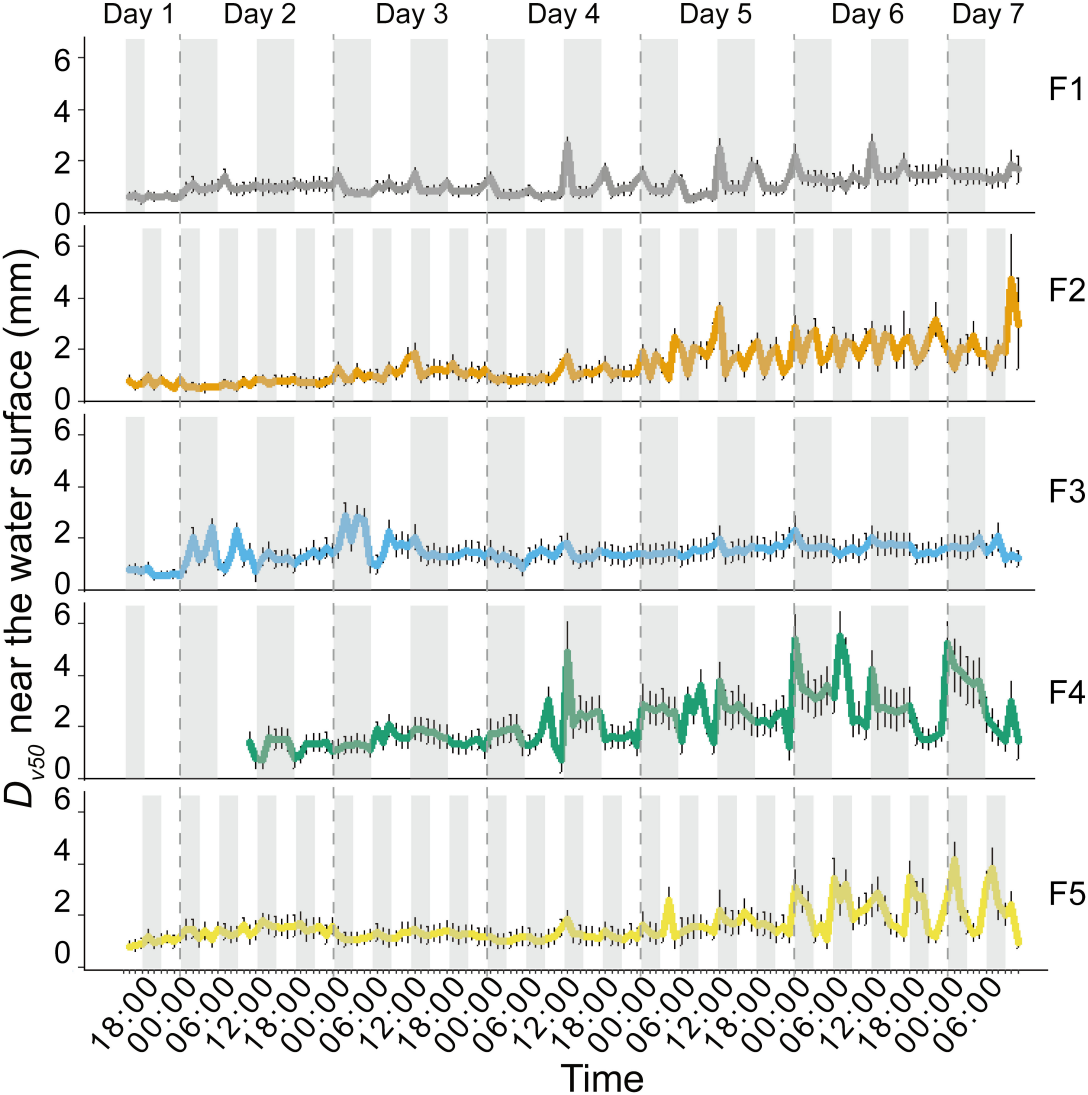
Temporal dynamics of the volume median diameter (*D_v50_*) of *Microcystis* colonies near the water surface (0 - 6 cm depth) in different experimental flumes (F1 – F5, see legend for color assignment). Symbols show mean values and error bars are standard deviations. The periods of high wind speed (wind disturbances) were represented with gray shading. The initial lack of data in F4 was due to a technical issue. F1: 6 h of high-magnitude (4.5 m^-1^) wind disturbance; F2: 3 h of high-magnitude wind disturbance; F3: 6 h of moderate-magnitude (3.8 m^-1^) wind disturbance; F4: 6 h of low-magnitude (1.5 m^-1^) wind disturbance; F5: 3 h of low-magnitude wind disturbance.

The colony size showed temporal trends during the 7-day experiment (**Figure 4**). In the upper layer near the water surface, *Dv*_50_ increased from the beginning to Day 7 by a factor of 2.4, 3.1, 2.4, 4.4 and 2.7 in F1- F5, respectively. During the increase of *Dv*_50_, we observed the peaks in *Dv*_50_ of *Microcystis* colonies near the water surface at times when the wind speed changed from low to high from Day 3 – Day 7 (**Figure 4**). Through visual observations, we found that such larger colonies suddenly appearing in the bulk water resulted from wind-induced entrainment of surface scum patches forming during preceding low wind periods.

**Figure 5:**
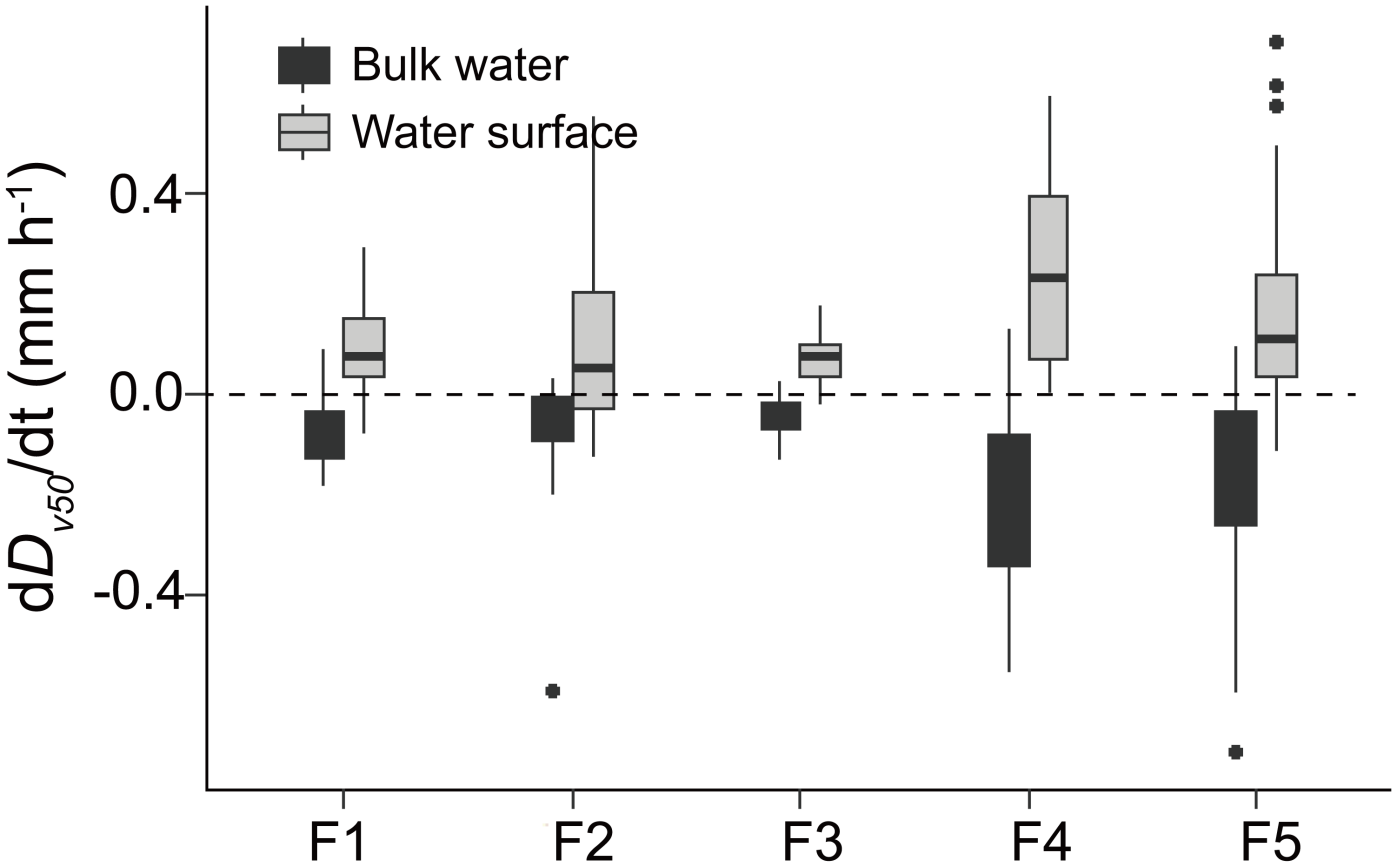
Rates of change in colony size (d*D_v50_*/dt) at the water surface (grey-filled box plots) and in the bulk water (black-colored box plots) in different flumes (F1 – F5). The dashed horizontal line indicates the d*D_v50_*/dt of 0 mm h^-1^. Significant differences between d*D_v50_*/dt at the water surface and in the bulk water were observedin all flumes (*t*-test, df=21 and 44, *p* < 0.001). F1: 6 h of high-magnitude (4.5 m^-1^) wind disturbance; F2: 3 h of high-magnitude wind disturbance; F3: 6 h of moderate-magnitude (3.8 m^-1^) wind disturbance; F4: 6 h of low-magnitude (1.5 m^-1^) wind disturbance; F5: 3 h of low-magnitude wind disturbance.

**Figure 6:**
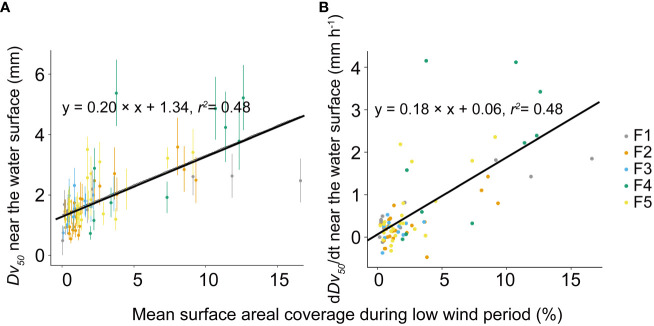
Relationship between the median diameter (*D_v50_*) of *Microcystis* colonies near the water surface at the onset of high wind disturbances and mean surface areal coverage during the low wind period before the disturbance **(A)**. Relationship between the rate of change in the colony median diameter (d*D_v50_*/dt) near the water surface at the onset of wind disturbances and mean surface areal coverage during low wind periods before the disturbance **(B)**. Symbols show mean values for different flumes (F1 – F5, see legend) and the error bars shown in **(A)** show the standard deviations. Both solid lines represent linear regressions based on the equations provided in the legend, with a significance level of *p* < 0.05. F1: 6 h of high-magnitude (4.5 m^-1^) wind disturbance; F2: 3 h of high-magnitude wind disturbance; F3: 6 h of moderate-magnitude (3.8 m^-1^) wind disturbance; F4: 6 h of low-magnitude (1.5 m^-1^) wind disturbance; F5: 3 h of low-magnitude wind disturbance.

The mean rate of change in colony size (d*Dv*_50_/dt) at the water surface and in the bulk water is -0.12 and 0.14 mm h^-1^, respectively. In each flume, the d*Dv*_50_/dt at the water surface is significantly higher than that in the bulk water (*p <*0.05, **Figure 5**). At the beginning of wind disturbances, both *Dv*_50_ and d*Dv*_50_/dt increased linearly with increasing SAC during the preceding low wind period (**Figure 6**).

The median diameter of *Microcystis* colonies (*Dv*_50_) was affected by the different wind conditions (**Figure 4**). *Dv*_50_ increased with decreasing magnitude of wind disturbances regardless of their duration (**Figure 4**). The response of *Dv*_50_ to the duration of the wind disturbance was dependent on its magnitude, with longer duration of high-magnitude wind disturbances leading to decreased *Dv*_50_ (F1 < F2, **Figure 4**), whereas *Dv*_50_ increased for longer durations of low-magnitude wind disturbances (F4 > F5, **Figure 4**). The order of mean *Dv*_50_ of *Microcystis* colonies near the water surface was F4>F5>F3>F2>F1 when combining all measurements (*p* < 0.05).

### 3.3 Hydrodynamics under different wind conditions

At the beginning of the experiment, the surface flow velocities increased nearly linearly with the applied wind speed resulting in equivalent wind speeds in the atmospheric boundary layer (*U*_10_) of 2.0 m s^-1^ under low wind speed conditions and 2.8 to 5.3 m s^-1^ during the simulated disturbances (**Table 1**).

**Table 1:** Wind speed measured at 2 cm above the water surface in the flumes and the corresponding equivalent wind speed in the atmospheric boundary layer at 10 m height (*U*_10_) derived from observed surface velocities at the beginning of the experiment using a wind factor of 0.01 (Wu, 1975).

Wind speed measured in the flumes (m s^-1^)	Calculated wind speed at 10 m height (m s^-1^)	Magnitudes of wind disturbance
0.5	2.0 ± 0.5	No wind disturbance
1.5	2.8 ± 0.2	Low-magnitude
3.8	3.9 ± 0.4	Intermediate-magnitude
4.5	5.3 ± 0.4	High-magnitude

**Figure 7:**
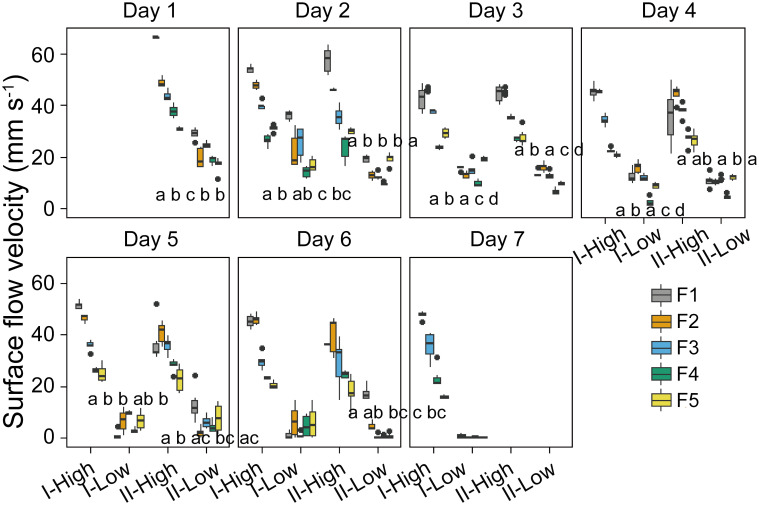
Temporal dynamics of the mean surface flow velocity in different stages (I and II) and experimental flumes (F1 – F5, see legend). High and Low represent periods of high wind speed (wind disturbances, 4.5 m s^-1^ for F1 and F2, 3.8 m s^-1^ for F3, 1.5 m s^-1^ for F4 and F5) and low wind speed (0.5 m s^-1^), respectively. Different panels denote the time (day) after the start of the experiment. Each box plot shows mean flow velocities observed in hourly video observations in the given stage in different flumes during the 7 days. Different lowercase letters indicate significant differences in flow velocity at different stages during low wind periods among different flumes, while the same letter, or the lack of a letter, indicates no significant differences. F1: 6 h of high-magnitude (4.5 m^-1^) wind disturbance; F2: 3 h of high-magnitude wind disturbance; F3: 6 h of moderate-magnitude (3.8 m^-1^) wind disturbance; F4: 6 h of low-magnitude (1.5 m^-1^) wind disturbance; F5: 3 h of low-magnitude wind disturbance.

The surface flow velocity and underwater flow velocities (at three depths) observed at a particular wind speed generally decreased from Day 1 to Day 7 (**Supplementary Figure S4**). The normalized flow velocity (defined as the ratio of flow velocity to wind speed) at the water surface and in the bulk water generally decreased linearly over time (*p* < 0.05, **Supplementary Figure S5**, **Supplementary Table S3**). In all flumes, the surface flow velocity and underwater flow velocities during low wind periods decreased to approximately 1 mm s^-1^ on Day 7 (**Figure 7**).

At the applied wind speed of 0.5 m s^-1^ (low wind periods), significant differences in surface flow velocity among different flumes were observed (*p <*0.05, **Figure 7**). No consistent dependence of surface flow velocity on the duration of the intermittent wind disturbance was observed, while surface flow velocity during low wind periods generally increased with increasing preceding magnitudes of wind disturbance: The order of surface flow velocity during low wind periods was F1>F3>F4, except for Day 6 stage I and Day 7. Unlike the surface flow velocity, the underwater flow velocities during low wind periods did not differ significantly between different flumes in most cases (**Supplementary Figure S6**).

### 3.4 Relationship between *Microcystis* and hydrodynamic parameters

Higher SAC was primarily observed at low surface flow velocities (< 5 mm s^-1^). However, it is noteworthy that SAC at moderate surface flow velocities (5-12 mm s^-1^) on Day 3 – Day 6 could also surpass some SAC at low surface flow velocities (**Supplementary Figure S7**). Neither *Dv*_50_ of colonies nor (d*Dv*_5_/dt) were linearly related to the root-mean-square velocities of colonies in the bulk water (*U_rms_*, calculated using Equation 1) when combining all measurements (**Supplementary Figure S8**). However, the proportion of d*Dv*_50_/dt being smaller than -1 mm h^-1^ (indicating colony disaggregation) increased with increasing *U_rms_*. Conversely, the proportion of d*Dv*_50_/dt larger than 0.5 mm h^-1^ (colony aggregation) decreased with increasing *U_rms_* when *U_rms_* exceeded 3 mm s^-1^and increased with increasing *U_rms_* when *U_rms_* was smaller than 3 mm s^-1^ (**Supplementary Figure S9**).

**Figure 8:**
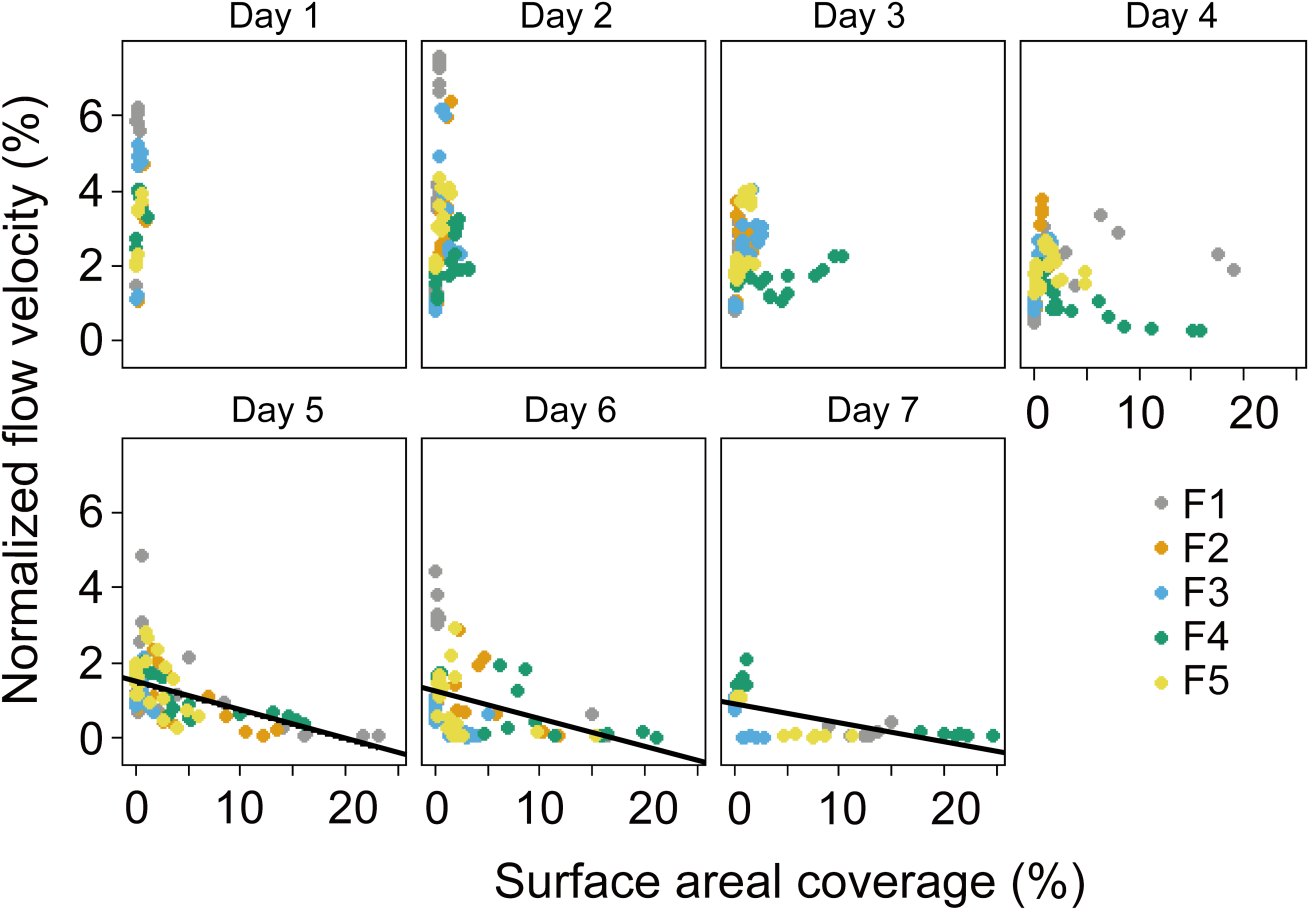
Normalized surface flow velocities (ratio of surface flow speed to wind speed) versus areal coverage of the water surface with algae. Symbols show mean values for different flumes F1 – F5, see legend. Different panels denote the time (day) during the experiment. No significant linear relationship was observed from Day 1 - 4 (*p* > 0.05), while significant linear relationship was observed from Day 5 to Day 7 (solid black lines, *p* < 0.05). F1: 6 h of high-magnitude (4.5 m^-1^) wind disturbance; F2: 3 h of high-magnitude wind disturbance; F3: 6 h of moderate-magnitude (3.8 m^-1^) wind disturbance; F4: 6 h of low-magnitude (1.5 m^-1^) wind disturbance; F5: 3 h of low-magnitude wind disturbance.

**Figure 9:**
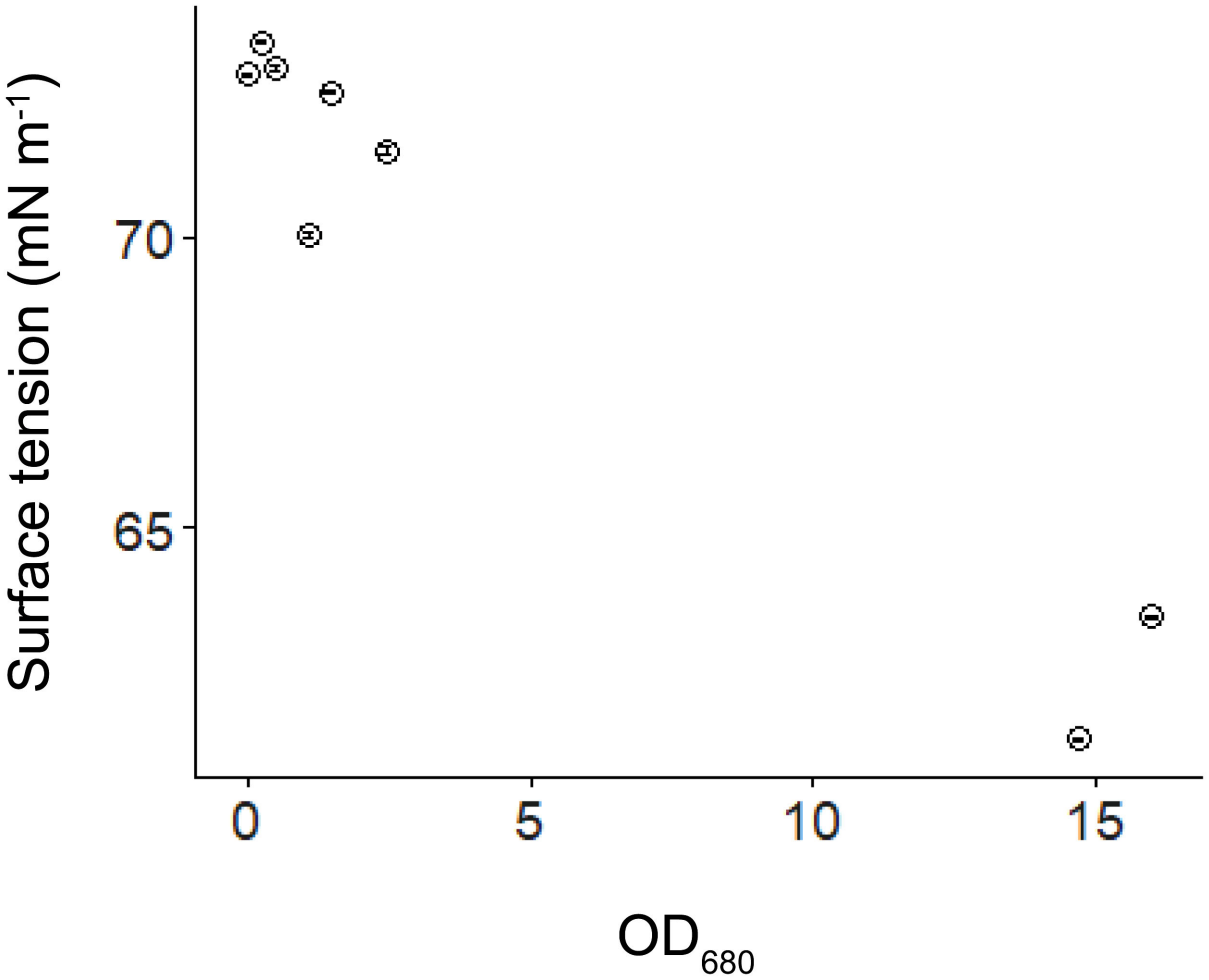
Water surface tension versus optical density of algal samples at 680 nm (OD_680_). Error bars show the standard deviation of replicated surface tension measurements (*n*=3).

The presence of *Microcystis* affected the hydrodynamic parameters: In the flumes experiment, from Day 1 to Day 4, no linear relationships between the normalized flow velocity and SAC were found, but from Day 5 to Day 7, the normalized flow velocity decreased with increasing SAC (*p* < 0.05, **Figure 8**). The results of our supplementary dilution experiment showed that the surface tension decreased with the increasing optical density of *Microcystis* (**Figure 9**).

## 4 Discussion

### 4.1 The response of colony size and scum of *Microcystis* to wind-generated hydrodynamics

Our experiments successfully reproduced the periodical formation and dispersion of *Microcystis* surface scum under intermittent wind disturbances (**Figure 2**). During low wind periods (0.5 m s^-1^), we observed gradually increased surface scum in all the flumes. Surprisingly, we found that scum was forming more rapidly following prolonged duration of high-magnitude wind disturbances compared to that following moderate magnitudes (F1, **Figure 2**). The differences in scum coverage in varying flumes can be attributed to the flow conditions, as they govern the migration of *Microcystis*. However, we found significant differences in flow velocities among flumes were only evident for the surface (**Supplementary Figure S6**; **Figure 7**), suggesting that the surface flow field likely played a pivotal role in modulating the response of scum formation to preceding wind disturbances.

Higher scum coverage can be observed at moderate surface flow velocities (5 - 12 mm s^-1^) compared to lower surface flow velocities in some cases (**Supplementary Figure S7**). This result implies potential mechanisms for surface scum formation under moderate flow velocities, considering that surface scum is typically associated with lower flow conditions (Wu et al., 2015). Webster and Hutchinson (1994) suggested the trapping mechanisms of colonies by viscous sublayer. A possible explanation we proposed is the strong vertical velocity gradient within the viscous sublayer, where the higher flow velocities near the water surface result in lower static pressure and a net upward lift force on the colonies, of which the magnitudes depend on the colony size and morphology. Although this process was not resolved in our study due to the lack of small tracer particles, we speculate that these complex interactions may account for the higher scum coverage observed at moderate flow velocities following a prolonged period (6 h) of high-magnitude wind disturbance. Future studies should include a more detailed characterization of the hydrodynamic conditions at the water surface (e.g., adding additional small seeding particles to perform particle image velocimetry) to better understand this mechanism.

The observed increase in surface scum also led to more frequent aggregations among colonies at the air-water interface. The developing scum significantly increased the colony size (**Figure 6**), thereby explaining the higher rates of change in colony size at the surface compared to in the bulk water (**Figure 5**). Given the limited attention to these aggregations, the underlying mechanism is unclear. Here we proposed capillary forces could be a driver for the formation of *Microcystis* aggregations at the water surface. In the vicinity of immersed buoyant particles, attractive forces arise due to micro-deformation of the air-water interface (capillary immersion force, Kralchevsky and Nagayama, 2000; Paunov et al., 1993). The common phenomenon that floating objects such as bubbles tend to clump together or cling to the sides of the container is driven by capillary forces (Vella and Mahadevan, 2005). We speculate that capillary forces partly increase the encounter rate of *Microcystis* at the water surface during prolonged periods of low wind speed, which promotes their accumulation and aggregation.

During wind disturbance, surface scums were dispersed throughout the bulk water, in which the hydrodynamics was governed by turbulent flow. Our study showed that moderate wind-generated turbulence promoted the aggregation of *Microcystis* into larger colonies, whereas high wind-generated turbulence favored their disaggregation (**Supplementary Figure S9**). This dual effect can be explained by the opposing effects of turbulence in increasing the collision frequency of cells and colonies and by increasing shear forces (Liu et al., 2019; O’Brien et al., 2004; Njobuenwu and Fairweather, 2018; Yao and Capecelatro, 2021). The latter likely limited the colony size in our experiments, which consistently decreased for increasing magnitudes of wind disturbances, regardless of disturbance duration (**Figure 4**). The duration of wind disturbances thus affects colony size by controlling the duration of interactions of colonies with turbulence, which may explain the magnitude-dependent effect of wind disturbance duration on colony size.

Periodic wind disturbances shifted the habitat of *Microcystis* between the air-water interface and the bulk water, during which the surface aggregation of *Microcystis* and the combination of their collision and disaggregation in the bulk water were alternating. In our experiments, such cycles constituted a positive feedback regulation between colony size and formation of surface scum, by which the size of suspended colonies and re-formation rate of scum continuously increased (**Figure 3**). It should be noted that colony size can also be affected by *Microcystis* growth (Xiao et al., 2018). However, the growth of *Microcystis* did not show notable differences among different flumes. In addition, the *D_v50_* changed in accordance with the wind conditions, with the peak in *D_v50_* occurring after the start of the disturbances (**Figure 4**), suggesting that the colony size dynamics were likely governed by the hydrodynamic conditions rather than by growth.

### 4.2 The effect of *Microcystis* surface scum on hydrodynamics under different wind conditions

We found a pronounced reduction in water surface tension with increasing *Microcystis* biomass with our supplementary experiment (**Figure 9**), which can have important consequences for the hydrodynamic processes at the air-water interface. We found that the normalized flow velocity decreased with increasing scum coverage at the later phase of our experiment (**Figure 8**), suggesting that the dense scum layer suppressed the momentum transfer from wind to water. At low wind speed, the reduction in momentum transfer can be explained by low water surface roughness, which is a consequence of low water surface tension (suppression of capillary waves, Wüest and Lorke, 2003). The increasing scum layer explained the decrease in water flow velocity generated by a given wind speed over time in our experiments (**Supplementary Figure S5**) and in a previous study (Wu et al., 2019). These findings implied that floating *Microcystis* can promote the formation and persistence of surface scum by altering surface tension and counteracting wind-driven mixing. Such reduced flow velocity caused by the presence of scum layers additionally contributed to the increasing re-formation rate of surface scum (**Figure 3**). The lack of direct measurements of surface tension in the flumes does not negate the surface tension-mediated hydrodynamic interactions between *Microcystis* and the air-water interface during the flume experiments, as indicated by the observed reduction in flow velocity.

The reduced surface tension caused by *Microcystis* colonies can also directly generate surface flows being directed towards regions of high surface tension (Marangoni effect (Roché et al., 2014; Vinnichenko et al., 2018), i.e., a spreading towards *Microcystis-*free regions. Although we could not directly observe this flow in our experiment as the Marangoni flow was masked by the wind-driven flow, it is ubiquitously present in environmental flows (Scriven and Sternling, 1960). Similar to the ‘soap boat’ (a well-known visualization of Marangoni convection), we propose that the lateral gradient of surface tension in the vicinity of initial scum patches can drive their horizontal surface drift and reshape their distributions in the absence of external force (e.g., weak wind forcing).

### 4.3 The environmental relevance and limitations of the experiment

We scaled the wind speeds measured in the flumes to representative wind velocities at a standard measurement height of 10 m (*U_10_*, see **Supplementary Table S3**). In our experiment, the low wind speed (*U_10_* of 2 m s^-1^) corresponds to the wind magnitude during weak wind periods, while the wind disturbances (*U_10_* of 2.8 – 5.3 m s^-1^) correspond to amplitudes of periodic wind forcing frequently observed over lakes (Saber et al., 2018; Fernández Castro et al., 2021). The chosen 6 h and 12 h periods of wind disturbances represent the lower and the upper range of field observations over lakes (Fernández Castro et al., 2021).

Surface scum formed during low wind periods and was dispersed during wind disturbances, which is in line with field investigation that showed an upper threshold of *U_10_* for scum persistence is 2.6 - 3 m s^-1^ (George and Edwards, 1976; Cao et al., 2006; Qi et al., 2018). Moreover, light-induced changes in the buoyancy of *Microcystis*, commonly observed in the field (Ibelings et al., 1991), were successfully reproduced (**Figure 2**). These observations further demonstrate the validity of our experimental design.

The water depth in our study was limited by the dimensions of the flume to 30 cm, which is smaller than the amplitude of vertical migration of *Microcystis* in natural lakes. Consequently, the formation rates of surface scum that we observed in the flumes may be higher than those found in the field. In addition, the low water depth restricted vertical mixing at high wind speeds, exposing *Microcystis* in the bulk water to higher irradiance than in deeper water. However, the focus of this study was on the hydrodynamic interactions of surface scum with the air-water interface, which is not expected to be linked to the scale of the experimental setup or to water depth. As the light intensity affects both the growth and the buoyancy production of *Microcystis*, we utilized relatively high irradiance levels, as they often persist in surface waters in the field. Initially, our experiments also included the configuration of the low duration (3 h) and median-magnitude (3.8 m s^-1^) wind disturbances. That data, however, had to be excluded due to technical issues with the flume operation. Future experiments should be conducted at higher resolutions (e.g., analyzing finer gradients of disturbance magnitude and duration) in order to provide a more comprehensive understanding of the partly non-linear interactions of wind with surface scum. Furthermore, buoyancy-driven migration could not be resolved in our measurements, as the flow velocity (generally exceeding 1 mm s^-1^) surpasses the floatation velocity of most colonies (< 1 mm s^-1^, Wallace et al., 2000).

Our study reveals the close connection between *Microcystis* dynamics and the air-water interface. The air-water interface emerges as a crucial habitat increasing colony size during scum formation (typically under low wind speeds), surpassing the impact of bulk water (**Figure 5**). Recent studies have shown that the wind has declined in some regions over the past few decades (Vautard et al., 2010; Ranjbar et al., 2022). Consequently, this study indicates that the projected decrease in wind speed under future climate conditions will result in larger colonies and facilitate the recovery of surface scum upon wind disturbance. Additionally, our finding of denser scum after prolonged periods of high wind speeds may have implications for early warning of blooms during windy conditions.

Our study also suggests that *Microcystis* reduces surface tension, likely through the excretion of surface-active substances. This process constitutes a dispersion-avoidance mechanism under wind conditions, allowing *Microcystis* to persist at well-lit depths. Furthermore, reduced surface tension potentially drives lateral convection, facilitating the expansion of *Microcystis* and mitigating competition for resources during weak wind periods. These processes may represent adaptive responses of *Microcystis* to wind stressors, suggesting that water surface tension, affected by *Microcystis*, may be an important mediator by which scum-forming cyanobacteria shape their habitat in favorable directions. Given the oversight of the aggregation of *Microcystis* at the air-water interface and their hydrodynamic interactions in current studies, it is important to consider these processes appropriately for a more accurate prediction of *Microcystis* scum dynamics in future climatic conditions.

## Data availability statement

The raw data supporting the conclusions of this article will be made available by the authors, without undue reservation.

## Author contributions

HW: Formal Analysis, Investigation, Writing – original draft. XW: Conceptualization, Funding acquisition, Writing – review & editing. LR: Methodology, Writing – review & editing. AL: Conceptualization, Funding acquisition, Methodology, Writing – review & editing.
