# Supplementary material for: Dynamics of Microcystis surface scum formation under different wind conditions: the role of hydrodynamic processes at the air-water interface
Source: Front Plant Sci. 2024 Mar 11;15:1370874. doi: 10.3389/fpls.2024.1370874 (PMC10961403; doi:10.3389/fpls.2024.1370874)
Supplement: Supplementary file 1 [file DataSheet_1.pdf]

Supplementary Material

Supplementary Figures and Tables

1. Supplementary Tables

Supplementary Table 1: The composition of the stock solution containing BG11 medium (100X, Blue-Green Medium).

Compound	Concentration [g L^{-1}]
$NaNO_3$ (sodium nitrate)	150
$K_2HPO_4 . 3H_2O$ (dipotassium phosphate)	3.14
$MgSO_4 .7H_2O$ (magnesium sulfate)	3.60
$CaCl_2.2H_2O$ (calcium chloride)	3.67
citric acid	0.56
Ferric ammonium citrate	0.60
EDTA (dinatrium-salt)	0.10
Na_2CO_3 (sodium carbonate)	2.00

Supplementary Table 2: The resolution and size of the field of view of the videos recorded by the cameras in the flume experiments.

Flume	Camera	Resolution (μm pixel^{-1})	Field of view (cm^2)
F1	Down-looking	39.7	7.6 × 4.3
	Upper side-looking	25.5	4.9 × 2.8
	Middle side-looking	22.4	4.3 × 2.4
	Bottom side-looking	24.5	4.7 × 2.6
F2	Down-looking	40.0	7.7 × 4.3
	Upper side-looking	22.4	4.3 × 2.4
	Middle side-looking	26.0	5.7 × 3.2
	Bottom side-looking	29.9	7.6 × 4.3
F3	Down-looking	39.6	4.5 × 2.5
	Upper side-looking	23.5	5.6 × 3.2
	Middle side-looking	29.4	6.0 × 3.7
	Bottom side-looking	31.2	7.6 × 4.3
F4	Down-looking	39.2	7.5 × 4.2
	Upper side-looking	24.8	4.8 × 2.7
	Middle side-looking	30.1	5.8 × 3.3
	Bottom side-looking	28.2	5.4 × 3.0
F5	Down-looking	39.0	7.5 × 4.2
	Upper side-looking	21.7	4.2 × 2.3
	Middle side-looking	26.8	5.1 × 2.9
	Bottom side-looking	29.0	5.6 × 3.1

Supplementary Table 3: Linear regression coefficients for the flow velocity normalized by wind speed (*y*) versus time during the experiment (*x* in days) observed at the water surface and in three different layers of the water column in the five experimental flumes (F1-F5). r^2 denotes the coefficient of determination of the regressions. Except for near surface in F3, middle layer in F1 and bottom in F2, the slopes were significantly different from zero (p <0.05).

	Surface	Near surface	Middle layer	Bottom
F1	y=-0.55*x+4.29<br>r^2=0.82	y=-0.08*x+1.31<br>r^2=0.70	y=-0.04*x+0.94<br>r^2=0.54	y=-0.02*x+0.73<br>r^2=0.74
F2	y=-0.37*x+3.12<br>r^2=0.93	y=-0.06*x+1.34<br>r^2=0.70	y=-0.07*x+1.40<br>r^2=0.67	y=-0.06*x+1.32<br>r^2=0.41
F3	y=-0.48*x+3.62<br>r^2=0.90	y=-0.10*x+1.30<br>r^2=0.56	y=-0.09*x+1.18<br>r^2=0.91	y=-0.09*x+1.01<br>r^2=0.82
F4	y=-0.36*x+3.04<br>r^2=0.79	y=-0.10*x+1.30<br>r^2=0.72	y=-0.09*x+0.94<br>r^2=0.85	y=-0.06*x+0.63<br>r^2=0.90
F5	y=-0.42*x+3.54<br>r^2=0.99	y=-0.10*x+1.08<br>r^2=0.73	y=-0.09*x+1.20<br>r^2=0.65	y=-0.11*x+1.07<br>r^2=0.96

2 Supplementary Figures

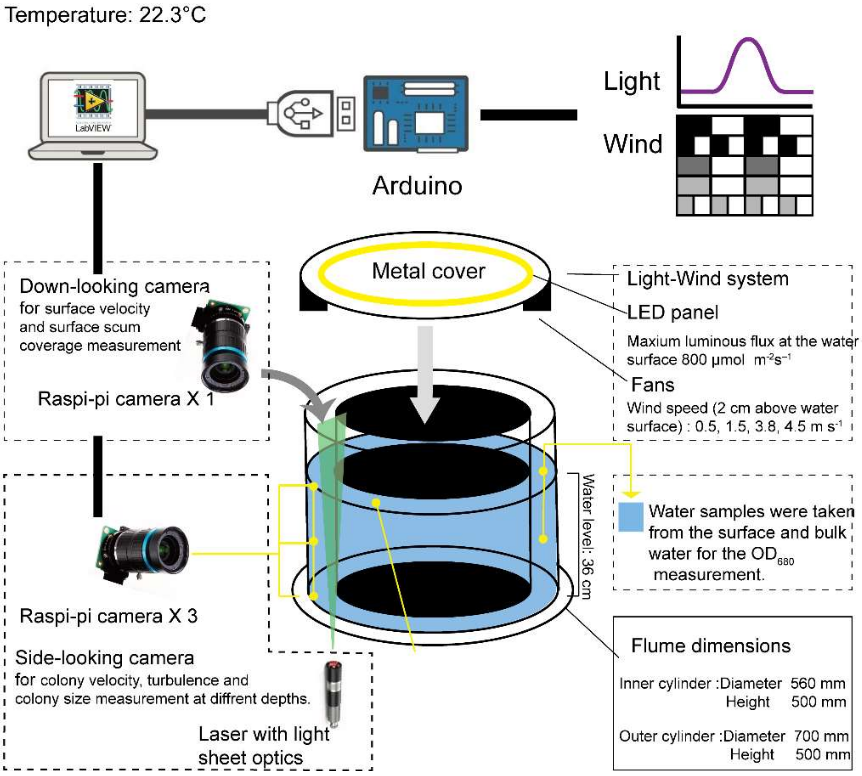

Supplementary Figure 1. Laboratory mesocosms with controlled environmental conditions including wind-generated turbulence and detailed characterization of *Microcystis* colony size and velocity. The water flows between the inner and outer cylinders of the annular flume. The flow is generated by wind, provided by two diagonally arranged fans (5 cm diameter, 12 V maximum supply voltage) above the water surface. Illumination of algae is provided by the five Light-Emitting Diode (LED) panels installed on the lid. Colonies at the air-water interface are observed by a downward-looking camera. In the bulk water, colonies are observed by three side-looking cameras and vertically arranged laser light sheet illumination. The light intensity and wind speed are controlled by an Arduino microcontroller. The microcontroller, all cameras, and the laser illumination for all five flumes are controlled by a central computer using a LabView (community edition, National Instruments, USA) program.

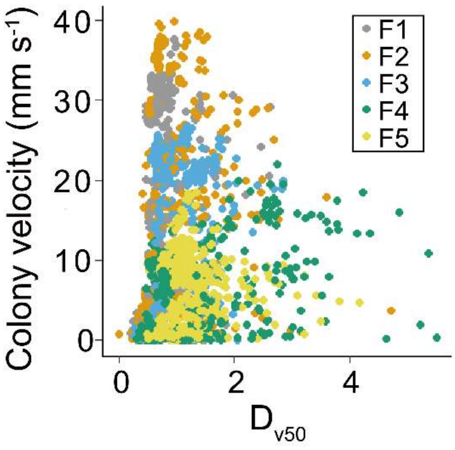

Supplementary Figure 2. The relationship between colony velocity and median volume diameter (D_{v50}) of *Microcystis* colonies.

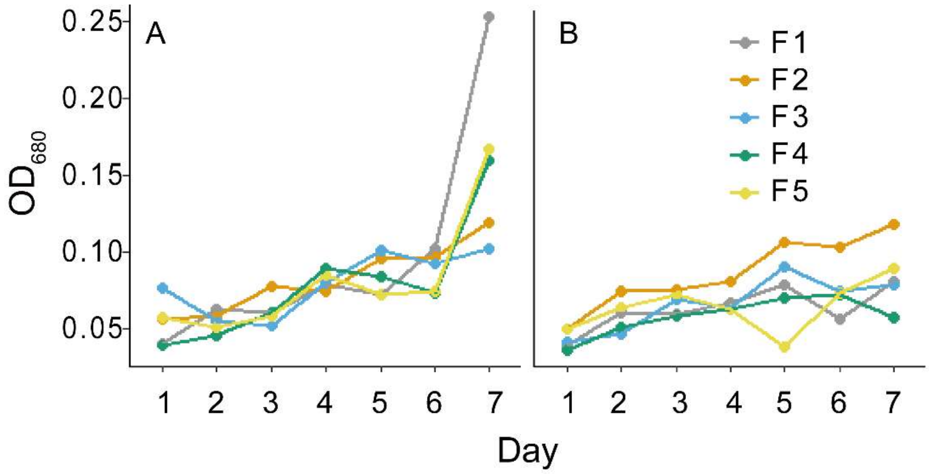

Supplementary Figure 3. The temporal dynamics of optical density at 680 nm (OD_{680}) of *Microcystis* samples in different flumes (see the color assignment) at the water surface (a) and in the middle layer in different flumes (b).

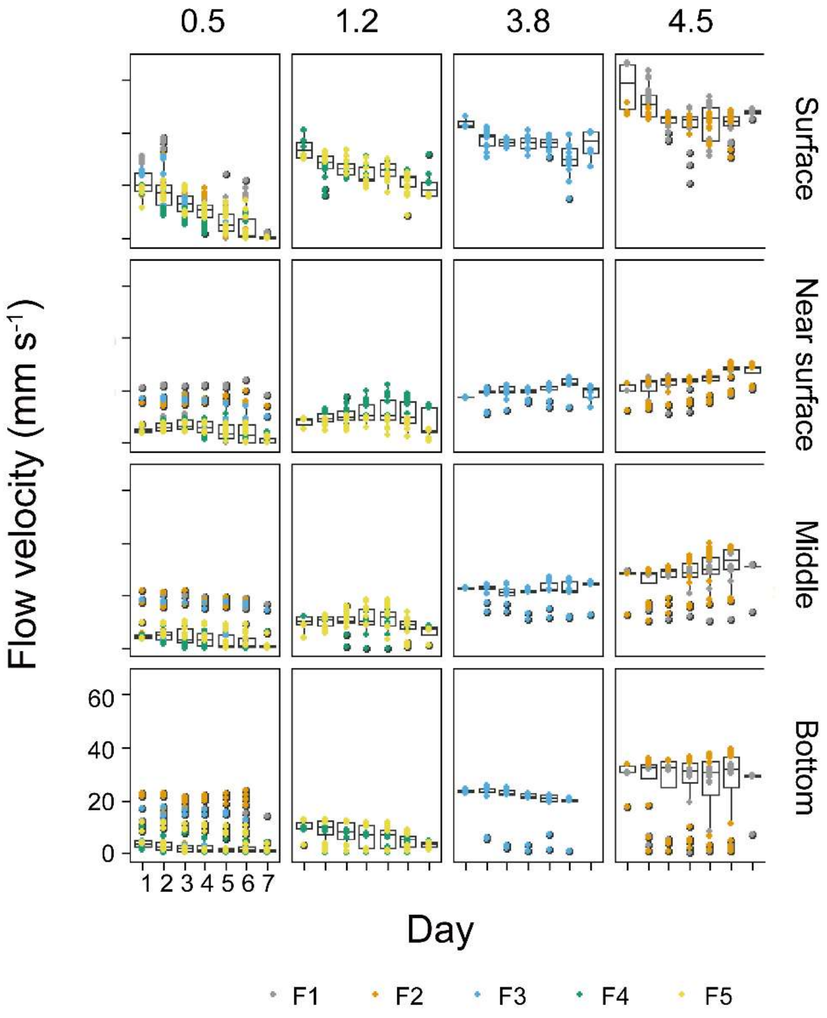

Supplementary Figure 4. Time series of flow velocity at different depths (rows) observed at different wind speed (columns, measured 2 cm above the water surface in the flumes) in different flumes (see legend for color assignment).

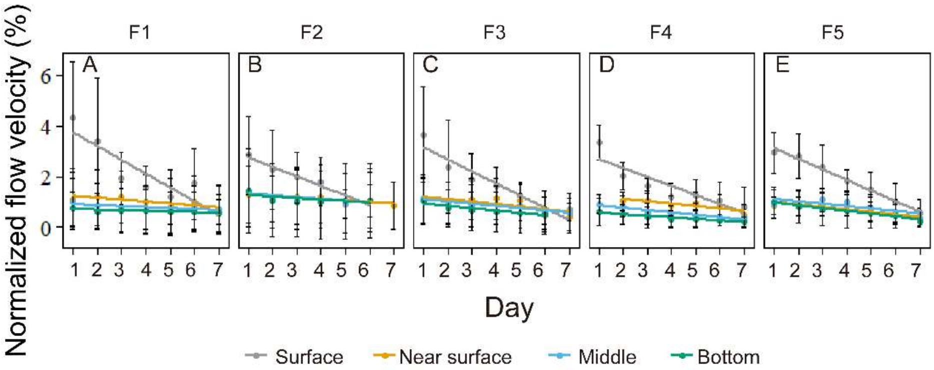

Supplementary Figure 5. Daily mean estimates of the normalized flow velocity (ratio of flow velocity to wind speed) at different depths (see legend for color assignment) to wind speed. Error bars show the standard deviation of the normalized flow velocity. The solid lines show linear regressions according to the equation provided (see Table.S2 for detailed equations and r^2). Except for near surface in F3, middle layer in F1 and bottom in F2, the slopes were significantly different from zero (p <0.05).

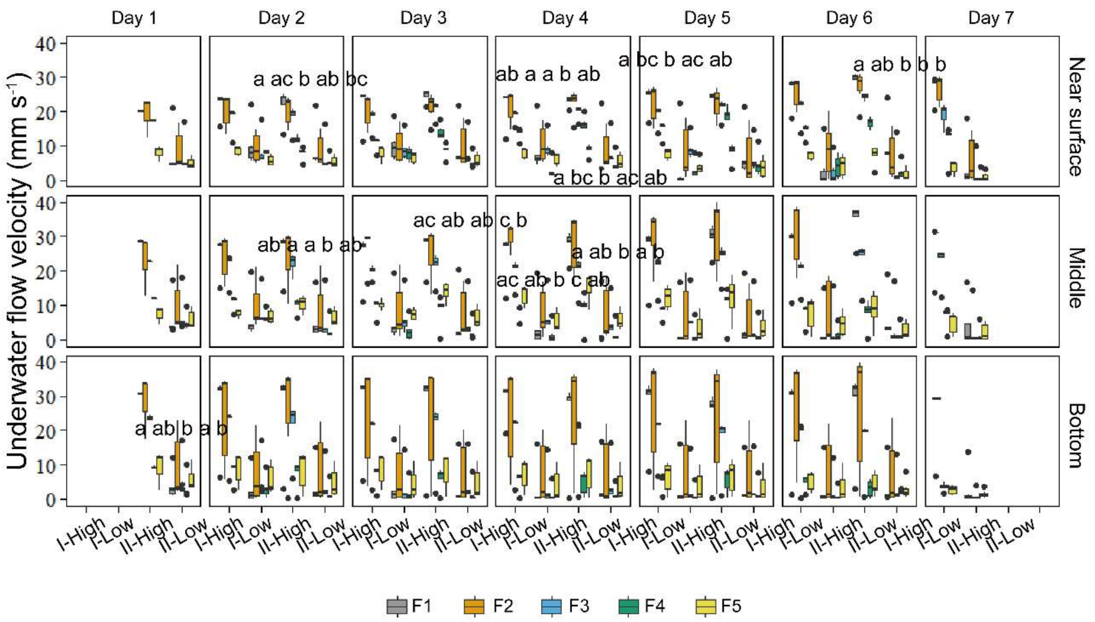

Supplementary Figure 6. Temporal dynamics of the mean underwater flow velocity at different water depth (rows) at different stages (I and II) and in different flumes (F1 – F5, see legend). High and Low represent the period of high wind speed (wind disturbances, 4.5 m s^{-1} for F1 and F2, 3.8 m s^{-1} for F3, 1.5 m s^{-1} for F4 and F5) and low wind speed (0.5 m s^{-1}), respectively. Each box plot shows mean flow velocities observed in hourly video observations at the given stage in different flumes during the 7 days. Different lowercase letters indicate significant differences in surface flow velocity at different stages during low wind periods among different flumes, while the same letter, or the lack of a letter, indicate no significant differences.

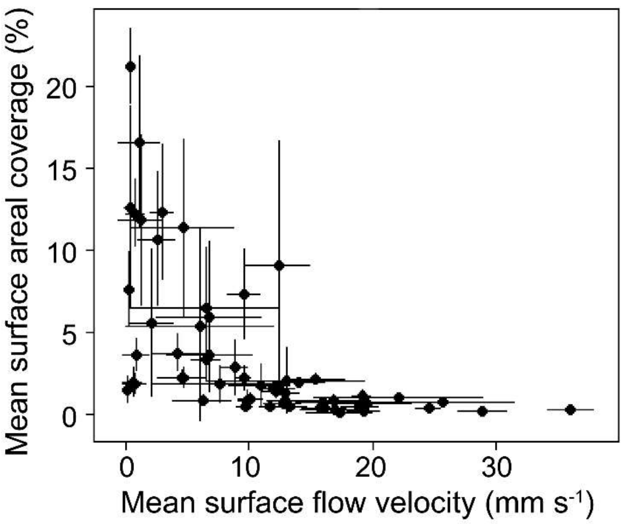

Supplementary Figure 7. Relationship between mean surface areal coverages and mean surface flow speed during low wind periods. Symbols show mean values and error bars indicate standard deviations.

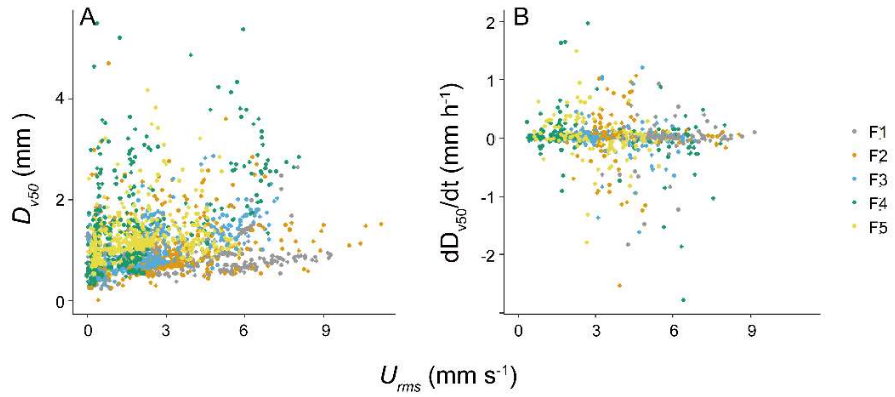

Supplementary Figure 8. The relationship between the median volume diameter (D_{v50}) and the root-mean-square of colony velocity (U_{rms}) (a), and between rate of change of colony size (dD_{v50}/dt) and U_{rms} (b). Different colour represent measurements in different flumes (see legend for color assignment).

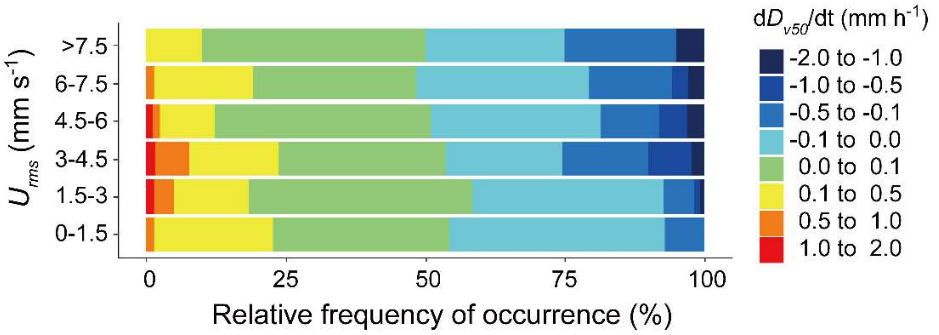

Supplementary Figure 9. Normalized frequency distributions of the mean rate of change of D_{v50} of *Microcystis* colonies (dD_{v50}/dt, see legend for color scaling) for different ranges of root-mean-square colony velocities (U_{rms}).
